# Association between polymorphisms in interleukins and oral lichen planus: A meta-analysis

**DOI:** 10.1097/MD.0000000000006314

**Published:** 2017-03-24

**Authors:** Quan Shi, Tong Zhang, Na Huo, Yang Huang, Juan Xu, Hongchen Liu

**Affiliations:** Institute of Stomatology, Chinese PLA General Hospital, Beijing, China.

**Keywords:** gene polymorphisms, interleukins, meta-analysis, oral lichen planus

## Abstract

**Background:**

More and more studies have suggested that single-nucleotide polymorphisms (SNPs) in interleukin (IL) genes are correlated with an increased risk of developing oral lichen planus (OLP). However, these results were inconsistent. Therefore, the aim of this meta-analysis is to retrieve and comprehensively analyze all related clinical studies to investigate the association of ILs gene polymorphisms with the OLP risk.

**Methods:**

PubMed, Embase, and the Cochrane Library were searched for eligible studies to evaluate the association between IL polymorphisms and the OLP. The odds ratios (ORs) and 95% confidence intervals (CIs) from each study were pooled to estimate the strength of the association. Statistical analyses were performed by using STATA software.

**Results:**

In all 6 studies, including 4 SNPs (IL6-174G/C, IL10-592C/A, IL10-819C/T, and IL10-1082G/A), 362 OLP patients and 622 non-OLP control subjects from five different countries were investigated. As for the IL6-174G/C, IL10-819C/T, and IL10-1082G/A, no evidence was found to support the association between SNP and OLP susceptibility in any genetic models. However, as for IL10-592C/A, a significant relationship between them was identified in all of comparison models (C vs A: OR = 0.724, 95% CI = 0.585–0.897, *P* = 0.003; CC vs AA: OR = 0.447, 95% CI = 0.276–0.722, *P* = 0.001; AC vs AA: OR = 0.585, 95% CI = 0.387–0.883, *P* = 0.011; CC+AC vs AA: OR = 0.544, 95% CI = 0.365–0.809, *P* = 0.003; CC vs AA+AC: OR = 0.715, 95% CI = 0.515–0.994, *P* = 0.046).

**Conclusion:**

With the presently available evidence, this meta-analysis fails to show the statistical associations between IL6-174G/C, IL10-819C/T, and IL10-1082G/A and OLP susceptibility in any genetic models. However, the A allele and AA genotype in IL10-592C/A polymorphism may increase the risk of OLP. In the future, more well-designed studies with larger sample sizes are needed.

## Abstract 2

Supplemental Digital Content is available in the text

## 1 Introduction

As one of the most common diseases of the oral mucosa, oral lichen planus (OLP) is affecting ∼0.5%–3% of the world population.^[1,2]^ It is a chronic inflammatory disease characterized by T-cell-mediated immune responses and abnormal epithelial keratinization cycles.^[3]^ OLP has been reported to carry a risk of malignant transformation to oral squamous cell carcinoma, thus it is defined as a premalignant condition.^[4,5]^ However, the etiology of OLP is unknown. It is widely accepted that genetic, environmental, immunological, endocrine, infectious, and psychological factors may be involved in the occurrence of OLP. ^[6]^

It is well known that interleukins (ILs) are a group of cytokines that play multiple functions in nearly all aspects of inflammation and immunity and participate in organism physiology and pathology regulation extensively, including OLP.^[7,8]^ So far, IL1, 2, 4, 5, 6, 8, 10, 12, 17, and 18 have been found to be the major ILs involved in the pathogenesis of OLP.^[9]^ ILs can be secreted by a multitude of cell types, including T lymphocytes.^[9]^ Considering that OLP is characterized by a dense subepithelial infiltration of lymphocytes, increased number of intraepithelial lymphocytes, and the degeneration of basal keratinocytes in histology, more and more researchers believe that ILs play a critical role in the pathogenesis and disease progression of OLP.^[3,10]^ Moreover, a growing number of literatures have proven that the level of ILs changes in lesions, saliva, serum, and peripheral blood mononuclear cells of OLP patients, hinting at the possibility of IL as diagnostic markers and therapeutic targets.^[11–14]^

Single-nucleotide polymorphisms (SNPs) in genes encoding for susceptibility factors may influence gene expression, protein function, and disease predisposition.^[15,16]^ More and more studies suggested that SNPs in interleukins genes, such as IL4 (−590T/C, −1098T/G), IL6 (−174G/C), IL10 (−592C/A, −819C/T, and −1082G/A), and IL18 (−137G/C, −607C/A), were correlated with development OLP or the severity of OLP.^[17–20]^ However, these results were mixed, inconclusive, and even inconsistent. Despite that Lu et al^[9]^ have briefly reviewed some of the research results on gene polymorphisms of several ILs in patients with OLP, specialized studies to quantitatively assess the presence and strength of the association between OLP and polymorphism of several ILs are still lacking.

Therefore, the aim of this meta-analysis is to identify and comprehensively analyze all related clinical studies to investigate the association of ILs gene polymorphisms with the OLP risk. Each type of IL polymorphism was analyzed if the study number was not <3. These results may provide more evidence for understanding the pathogenesis and progression of OLP, as well as provide a pathological basis for clinicians in the determination of further treatment and prognosis.

## 2 Methods

### 2.1 Identification of eligible studies

We searched PubMed, Embase, and the Cochrane Library for related studies without any restriction on February 3, 2017. The combination of the following keywords was used: “oral lichen planus OR lichen planus, oral” and “interleukins OR interleukin”. In addition, the reference lists given in the related articles and reviews were also considered for eligible studies. These results were independently assessed by two reviewers (SQ and ZT) according to the inclusion criteria, and any disagreement was resolved through discussion with a third reviewer (LHC). Ethical approval and informed consent were not required as this study was based on previously published studies and had no direct patient contact or influences on patient care.

### 2.2 Inclusion and exclusion criteria

In this meta-analysis, all available clinical studies evaluating the association between ILs polymorphisms and the OLP were included. The studies must conform to the following criteria to be eligible: clinical studies focusing on association between ILs polymorphisms and the OLP risk; containing detailed genotype data could be obtained to calculate the odds ratios (ORs) and 95% confidence intervals (CIs); the OLP patients and control subjects are described and confirmed clearly; for a single type of SNP of ILs, only when the number of study was not less than 3, would the quantitative analysis will be performed; otherwise, the studies were excluded from the quantitative analysis. The exclusion criteria were as follow: animal studies or in vitro studies; reviews, case reports, or comments; studies without available genotype data (including frequencies of alleles or genotypes in case and control groups) that could be extracted to estimate the OR value and 95% CI; family-based studies of pedigrees.

### 2.3 Data extraction and quality assessment

The following information was extracted from each included study: author, year of publication, country, design of study, clinical form of OLP, characteristics of the subjects (including the number of patients in groups, age, and gender), the type of IL and the SNPs, and genotype frequency in cases and controls. This process was independently performed by two reviewers (SQ and ZT).

A methodological quality assessment scale (Table S1) adopted from previous publications was used to assess the quality of the included studies.^[21,22]^ In this assessment scale, representativeness of cases, source of controls, sample size, quality control of genotyping methods, and Hardy–Weinberg equilibrium (HWE) were used to appraise the methodological quality of the included studies, with a maximum of 10 points for each study. The scores of 0 to 4, 5 to 7, and 8 to 10 indicated poor, moderate, and good study quality, respectively.

### 2.4 Statistics analysis

Statistical analyses were performed by using STATA software (Version 12.0; Stata Corp, College Station, TX). ORs and 95% CIs were calculated to measure the strength of the association between IL polymorphisms and OLP risk. Pooled ORs were performed for allelic comparison (IL6-174G/C: G vs C; IL10-592C/A: C vs A; IL10-819C/T: C versus A; IL10-1082G/A: G vs A), homozygote model (IL6-174G/C: GG vs CC; IL10-592C/A: CC vs AA; IL10-819C/T: CC vs TT; IL10-1082G/A: GG vs AA), heterozygote model (IL6-174G/C: CG vs CC; IL10-592C/A: AC vs AA; IL10-819C/T: TC vs TT; IL10-1082G/A: AG vs AA), dominant model (IL6-174G/C: GG+CG vs CC; IL10-592C/A: CC+AC vs AA; IL10-819C/T: CC+TC vs TT; IL10-1082G/A: GG+AG vs AA), and recessive model (IL6-174G/C: GG vs CG+CC; IL10-592C/A: CC vs AC+AA; IL10-819C/T: CC vs TC+TT; IL10-1082G/A: GG vs AG+AA), respectively. HWE was evaluated for each study by the chi-square test in control groups, and *P* < 0.05 was considered a significant departure from HWE. Statistical heterogeneity between studies was tested using *I*^2^ statistics. A fixed effects model was used if *I*^2^<50%. *I*^2^> 50% was considered to be substantial heterogeneity and the random effects was used.

## 3 Results

### 3.1 Summary of the included studies

**Figure 1:**
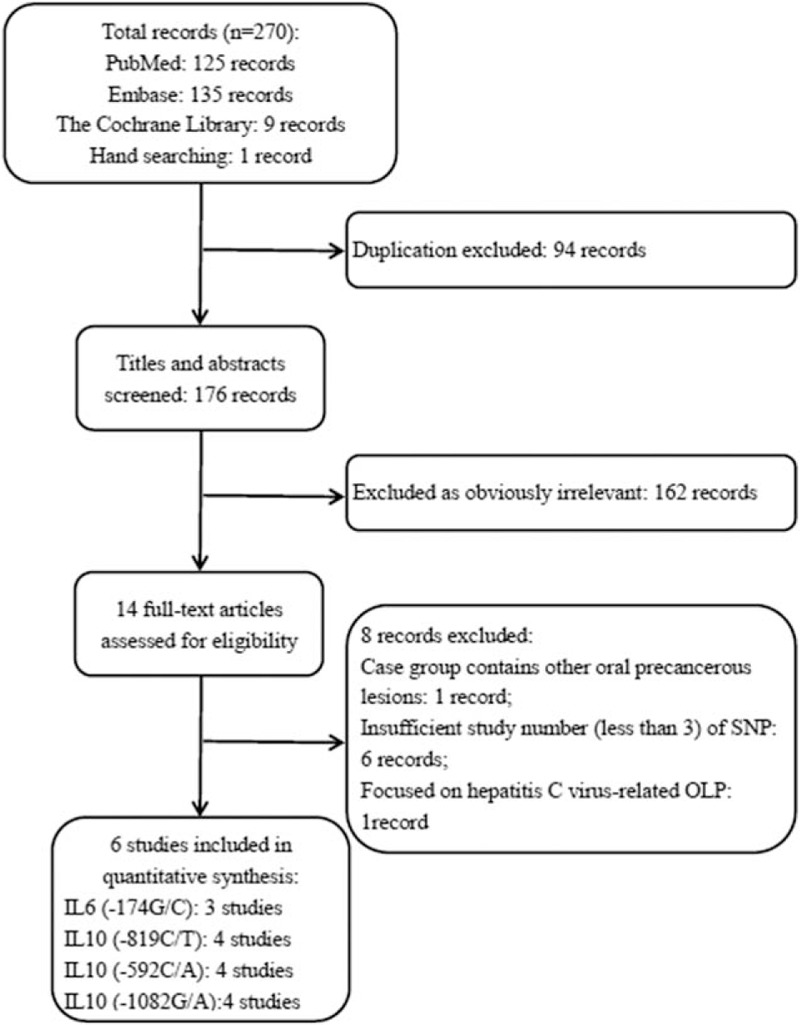
Flow diagram for the selection of studies. IL = interleukin, OLP = oral lichen planus, SNP = single-nucleotide polymorphism.

In total 270 potentially relevant articles were retrieved from PubMed, Embase, the Cochrane Library, and by hand searching according to our search strategies. A flow diagram of search process and results of the included studies are shown in Fig. 1. After the titles and abstracts were read, 162 articles not related to our focused topic were excluded according to the above inclusion criteria, leaving 14 articles for further full-text review. Then, one study^[16]^ was excluded because the case group contains other oral precancerous lesions; one study^[23]^ aimed to explore whether cytokine polymorphisms may influence the susceptibility to hepatitis C virus-related OLP; 6 studies were also excluded from the quantitative analysis because the total number of study related to the SNP of ILs they researched was <3. The total number of studies related to the SNP of ILs is shown in Table S2. Eventually, 6 studies^[18–20,24–26]^ including 4 SNPs (IL6-174G/C, IL10-592C/A, IL10-819C/T, and IL10-1082G/A) meeting the inclusion criteria were included for the ultimate quantitative analysis. All of the SNPs of ILs which were not analyzed in this meta-analysis were concluded in qualitatively in Table S2.

The design of the 6 included studies was case–control studies, and the publication dates ranged from 2004 to 2016. Of these included studies, a total of 362 OLP patients and 622 non-OLP control subjects from 5 different countries were covered. Three of 6 studies^[18,25,26]^ focused on the SNP of IL6-174G/C, whereas 4 of 6 studies^[19,20,24–26]^ focused on IL10-592C/A, IL10-819C/T, and IL10-1082G/A, respectively. The characteristics of the included studies, including the *P* value for HWE and quality assessment are summarized in Table 1 and S3.

**Table 1:**
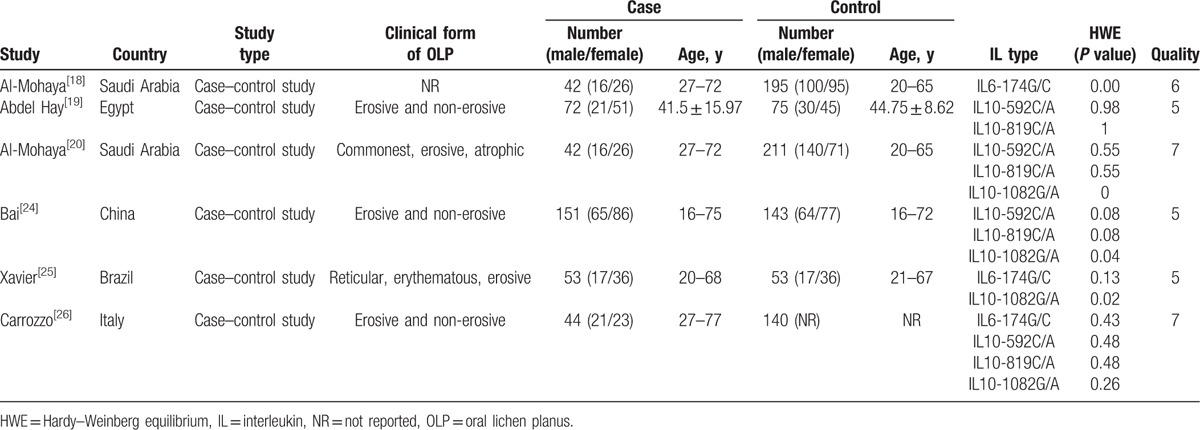
Characteristics of studies included in the meta-analysis.

### 3.2 Meta-analysis results

#### 3.2.1 IL6-174G/C

**Figure 2:**
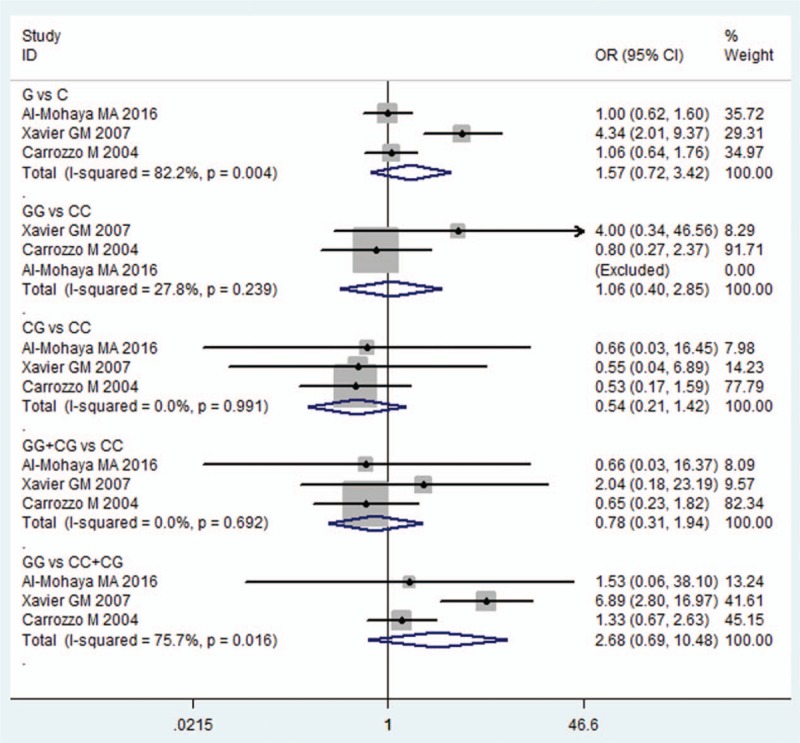
Forest plot of the IL6-174G/C polymorphism and OLP risk in all comparison models. CI = confidence interval, IL = interleukin, OLP = oral lichen planus, OR = odds ratio.

The meta-analysis of IL6-174G/C was based on 3 studies,^[18,25,26]^ and statistical heterogeneity between them was present in allelic model (*I*^2^ = 82.2%) and recessive model (*I*^2^ = 75.7%). Therefore, random effects model was applied in these analyses. For the overall test of allelic comparison (G vs C), the pooled OR was 1.570 (95% CI = 0.721–3.421, *P* = 0.256) and there was no significant association being identified (Fig. 2 and Table 2). In addition, no significant association was found between genotype models in IL6-174G/C polymorphism and OLP (GG vs CC: OR = 1.064, 95% CI = 0.396–2.855, *P* = 0.902; CG vs CC: OR = 0.540, 95% CI = 0.206–1.415, *P* = 0.210; GG+CG vs CC: OR = 0.782, 95% CI = 0.314–1.945, *P* = 0.597; GG vs CC+CG: OR = 2.685, 95% CI = 0.688–10.475, *P* = 0.155; Fig. 2 and Table 2).

**Table 2:**
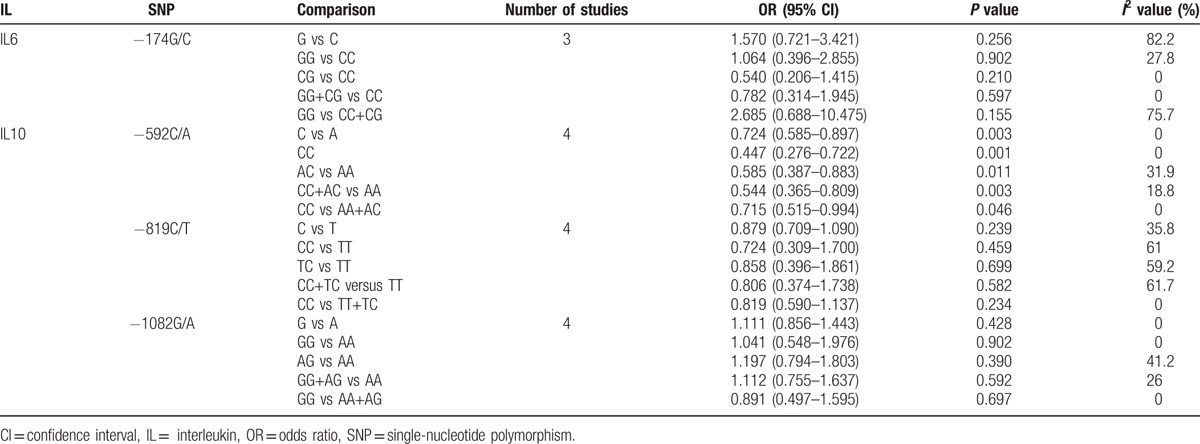
Summary of polled ORs in the meta-analysis.

#### 3.2.2 IL10-592C/A

**Figure 3:**
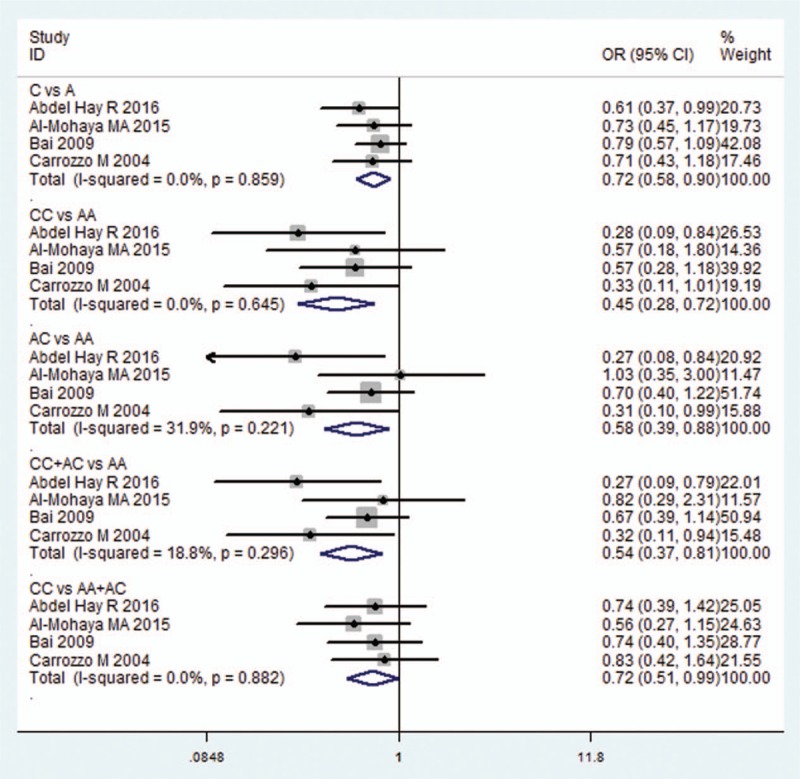
Forest plot of the IL10-592C/A and OLP risk in all comparison models. CI = confidence interval, IL = interleukin, OLP = oral lichen planus, OR = odds ratio.

Four studies^[19,20,24,26]^ researched the association of −592C/A in the IL10 gene between OLP patients and control subjects. Because the statistical heterogeneity between them was low (all *I*^2^ value were <50%), the fixed effects model was applied. A significant decreased risk of OLP was observed in all models (C vs A: OR = 0.724, 95% CI = 0.585–0.897, *P* = 0.003; CC vs AA: OR = 0.447, 95% CI = 0.276–0.722, *P* = 0.001; AC vs AA: OR = 0.585, 95% CI = 0.387–0.883, *P* = 0.011; CC+AC vs AA: OR = 0.544, 95% CI = 0.365–0.809, *P* = 0.003; CC vs AA+AC: OR = 0.715, 95% CI = 0.515–0.994, *P* = 0.046; Fig. 3 and Table 2).

#### 3.2.3 IL10-819C/T

**Figure 4:**
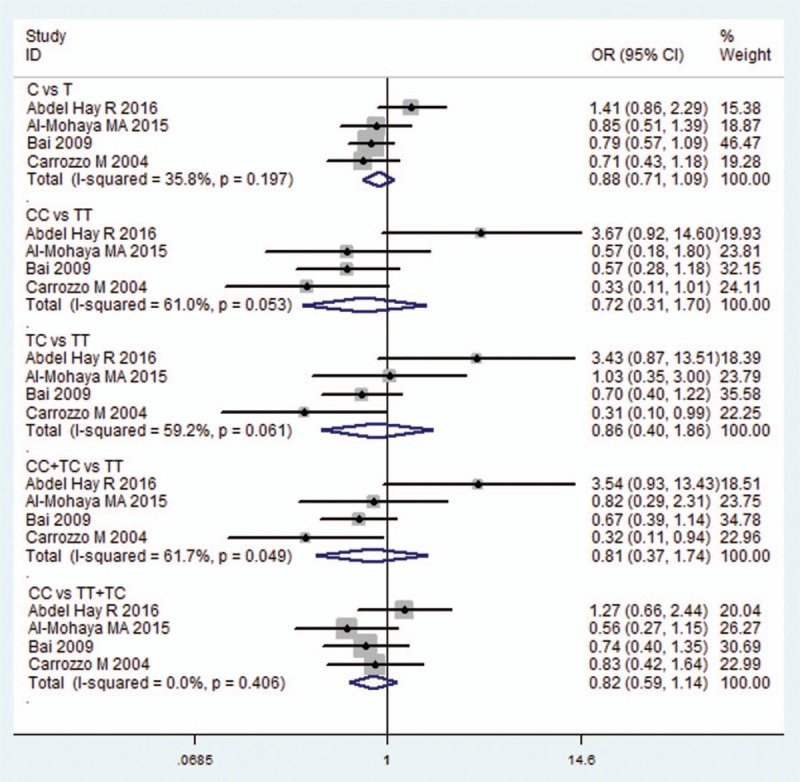
Forest plot of the IL10-819C/T and OLP risk in all comparison models. CI = confidence interval, IL = interleukin, OLP = oral lichen planus, OR = odds ratio.

Meta-analysis of the ORs between the OLP susceptibility and IL10-819C/T was performed in 4 included studies,^[19,20,23,25]^ which have reported available genotype data to calculate the OR and 95% CI. In the allelic model and recessive model, *I*^2^value was 35.8% and 0%, respectively, hence a fixed effects model was applied, whereas for the others, the random effects model was applied. However, no significant association was found in each comparison (C vs T: OR = 0.879, 95% CI = 0.709–1.090, *P* = 0.239; CC vs TT: OR = 0.724, 95% CI = 0.309–1.700, *P* = 0.459; TC vs TT: OR = 0.858, 95% CI = 0.396–1.861, *P* = 0.699; CC+TC vs TT, OR = 0.806, 95% CI = 0.374–1.738, *P* = 0.582; CC v TT+TC: OR = 0.819, 95% CI = 0.590–1.137, *P* = 0.234; Fig. 4 and Table 2).

#### 3.2.4 IL10-1082G/A

**Figure 5:**
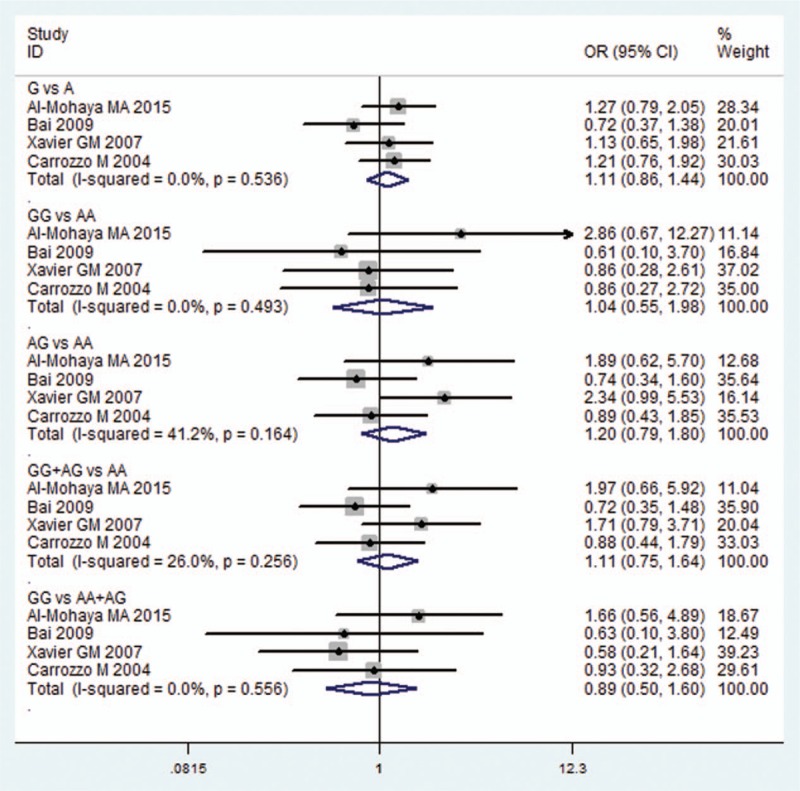
Forest plot of the IL10-1082G/A and OLP risk in all comparison models. CI = confidence interval, IL = interleukin, OLP = oral lichen planus, OR = odds ratio.

Four studies^[20,24–26]^ researched the association between OLP susceptibility and IL10-1082G/A compared with control subjects. The fixed effects model was applied in the overall comparison because of the low statistical heterogeneity in each model. No significant association was found in each comparison (G vs A: OR = 1.111, 95% CI = 0.856–1.443, *P* = 0.482; GG vs AA: OR = 1.041, 95% CI = 0.548–1.976, *P* = 0.902; AG vs AA: OR = 1.197, 95% CI = 0.794–1.803, *P* = 0.390; GG+AG vs AA: OR = 1.112, 95% CI = 0.755–1.637, *P* = 0.592; GG v AA+AG: OR = 0.891, 95% CI = 0.497–1.595, *P* = 0.697; Fig. 5 and Table 2).

## 4 Discussion

ILs are potent immunomodulators and proinflammatory cytokines with a broad range of biological activities in immune regulation, inflammation, hematopoiesis, and oncogenesis.^[7,8]^ More and more studies have found that ILs, including their variants, play a critical role in the occurrence, development, and treatment of OLP.^[9]^

In the present study, our purpose was to determine whether there was an association between IL SNPs and susceptibility to OLP. Six case-control studies and 4 SNPs of IL (IL6–174G/C, IL10-592C/A, IL10-819C/T, and IL10-1082G/A) meeting the inclusion criteria were included for the ultimate quantitative analysis. According to the results of meta-analysis, as for the IL6-174G/C, IL10-819C/T, and IL10-1082G/A, no evidence was found for the association between SNP and OLP susceptibility in any genetic models. However, as for association between IL10-592 C/A and OLP susceptibility, a positive relationship between them was identified. Moreover, the A allele and AA genotype in the IL10-592C/A polymorphism were significantly associated with an increased risk of OLP.

IL6, mainly produced by antigen-presenting cells (APCs),^[27]^ involved in OLP development may include T-cell growth and differentiation, apoptosis of keratinocytes from the basal layer and epithelial proliferation, resulting in hyperplasia.^[28,29]^ Besides, there was evidence that the level of IL6 was elevated in saliva and serum in OLP patients, especially in the erosive form of OLP.^[29,30]^ The results of our meta-analysis failed to find an association between IL6-174G/C and OLP susceptibility in any genetic models. This finding was consistent with those of Al-Mohaya et al^[18]^ and Carrozzo et al.^[26]^ However, Xavier et al^[25]^ found that IL6-174G/C homozygous genotypes were significantly more often detected in OLP patients and were associated with an increased risk of OLP development (OR = 6.89). Despite genotypes from the study of Al-Mohaya et al^[18]^ deprived from HWE, the results were stable after omitting this study (data were not shown).

As a cytokine secreted by macrophages, monocytes, and T lymphocytes, IL10 is thought to be involved in the pathogenesis of OLP.^[9,31]^ IL10 exhibits diverse functions in different T lymphocytes (such as T helper cell (Th) 1, Th2, and cluster of differentiation 4+ T lymphocytes), and high levels of serum and saliva IL10 were also found in the OLP patients. In this meta-analysis, the associations between 3 IL10 SNPs (IL10-592C/A, IL10-819C/T, and IL10-1082G/A) and the susceptibility of OLP were investigated. As for the SNP of IL10-819C/T and IL10-1082G/A, the results of our meta-analysis indicated that these 2 SNPs were not associated with the susceptibility of OLP, whereas a positive relationship between IL10-592C/A and OLP was identified in all of the models and our results indicated that the A allele and AA genotype was significantly associated with an increased risk of OLP as compared to the C allele. These results are similar to those of Abdel Hay et al^[19]^, which found that IL10-592 AA genotypes and IL10-592 A allele might contribute to the susceptibility of OLP. However, Al-Mohaya et al,^[20]^ Bai et al,^[24]^ and Carrozzo et al^[26]^ found that the frequencies of alleles and genotypes of these SNPs in the *IL10* gene did not differ significantly between OLP patients and control subjects. We should point out that these 3 studies^[20,24,25]^ focusing on the IL10-1082G/A were deprived from HWE through our test (Table 1), therefore the results of this SNP should to be treated cautiously.

The observed heterogeneity in some comparisons models and the inconsistent conclusions may be attributed to several factors. First, the included studies focused on different races, while the relationship between SNPs and diseases may be influenced by the race. Among the 6 included studies, three focused on the Asians,^[18,20,24]^ the others were focused on the Europeans,^[26]^ Africans,^[19]^ and Brazilians,^[25]^ respectively. Second, as mentioned above, several factors have been proposed for the etiology of OLP. However, the included studies matched or adjusted for different types and numbers of the confounding factors. Third, some studies were deprived from HWE. One was for IL6-174G/C^[18]^ and three for IL10-1082G/A.^[20,24,25]^ Therefore, well-designed studies with larger and well-matched samples were needed in the future.

To our knowledge, this is the first meta-analysis to estimate the association between susceptibility of OLP and SNP of ILs by quantitative analysis. We not only researched electronic databases to identify potential interests, but also manually examined reference lists from relevant studies. The methodological quality assessment scale was used to evaluate each of the included studies and none of them had low quality. Nevertheless, in spite of our efforts to explore the possible relationship between the SNPs of ILs and OLP risk, we have to acknowledge a number of limitations in this study. First, the number of studies included in our meta-analysis was limited. Second, heterogeneity was detected in some genetic models, which might partially influence the results. Third, because of the limited number of studies, publication bias analysis and the subgroup analysis of the included studies were not performed.

## 5 Conclusions

In conclusion, this meta-analysis fails to show statistical associations between IL6-174G/C, IL10-819C/T, and IL10-1082G/A, and OLP susceptibility in any genetic models. However, the results also indicate that the A allele and AA genotype in IL10-592C/A polymorphism are significantly associated with an increase of OLP susceptibility. However, the results should be interpreted with caution considering the limitations of the included studies and this meta-analysis. Well-designed studies with larger sample sizes and multiple ethnicities are required to strengthen the results of the present study.

## Supplementary Material

Supplemental Digital Content

## References

[1] McCartan BE Healy CM The reported prevalence of oral lichen planus: a review and critique. J Oral Pathol Med 2008;37:447–53. 18624932 10.1111/j.1600-0714.2008.00662.x

[2] Belfiore P Di Fede O Cabibi D Prevalence of vulval lichen planus in a cohort of women with oral lichen planus: an interdisciplinary study. Brit J Dermatol 2006;155:994–8. 17034531 10.1111/j.1365-2133.2006.07480.x

[3] Roopashree MR Gondhalekar RV Shashikanth MC Pathogenesis of oral lichen planus—a review. J Oral Pathol Med 2010;39:729–34. 20923445 10.1111/j.1600-0714.2010.00946.x

[4] Eisen D The clinical features, malignant potential, and systemic associations of oral lichen planus: a study of 723 patients. J Am Acad Dermatol 2002;46:207–14. 11807431 10.1067/mjd.2002.120452

[5] Lozada-Nur F Miranda C Oral lichen planus: epidemiology, clinical characteristics, and associated diseases. Semin Cutan Med Surg 1997;16:273–7. 9421218 10.1016/s1085-5629(97)80016-8

[6] Nogueira PA Carneiro S Ramos-e-Silva M Oral lichen planus: an update on its pathogenesis. Int J Dermatol 2015;54:1005–10. 26147778 10.1111/ijd.12918

[7] Brocker C Thompson D Matsumoto A Evolutionary divergence and functions of the human interleukin (IL) gene family. Hum Genomics 2010;5:30–55. 21106488 10.1186/1479-7364-5-1-30 PMC3390169

[8] Fietta P Costa E Delsante G Interleukins (ILs), a fascinating family of cytokines. Part I: ILs from IL-1 to IL-19. Theor Biol Forum 2014;107:13–45. 25936211

[9] Lu R Zhang J Sun W Inflammation-related cytokines in oral lichen planus: an overview. J Oral Pathol Med 2015;44:1–4. 24329772 10.1111/jop.12142

[10] Sugerman PB Savage NW Walsh LJ The pathogenesis of oral lichen planus. Crit Rev Oral Biol Med 2002;13:350–65. 12191961 10.1177/154411130201300405

[11] Zhang Y Liu W Zhang S Salivary and serum interleukin-18 in patients with oral lichen planus: a study in an ethnic Chinese population. Inflammation 2012;35:399–404. 21484426 10.1007/s10753-011-9327-3

[12] Dan H Liu W Wang J Elevated IL-10 concentrations in serum and saliva from patients with oral lichen planus. Quintessence Int (Berlin, Germany: 1985) 2011;42:157–63. 21359250

[13] Liu WZ He MJ Long L Interferon-gamma and interleukin-4 detected in serum and saliva from patients with oral lichen planus. Int J Oral Sci 2014;6:22–6. 24158143 10.1038/ijos.2013.74 PMC3967304

[14] Pekiner FN Demirel GY Borahan MO Cytokine profiles in serum of patients with oral lichen planus. Cytokine 2012;60:701–6. 22995209 10.1016/j.cyto.2012.08.007

[15] Nachman MW Single nucleotide polymorphisms and recombination rate in humans. Trends Genet 2001;17:481–5. 11525814 10.1016/s0168-9525(01)02409-x

[16] Hsu HJ Yang YH Shieh TY Role of cytokine gene (interferon-gamma, transforming growth factor-beta1, tumor necrosis factor-alpha, interleukin-6, and interleukin-10) polymorphisms in the risk of oral precancerous lesions in Taiwanese. Kaohsiung J Med Sci 2014;30:551–8. 25458044 10.1016/j.kjms.2014.09.003 PMC11916204

[17] Bai J Lin M Zeng X Association of polymorphisms in the human IFN-gamma and IL-4 gene with oral lichen planus: a study in an ethnic Chinese cohort. J Interferon Cytokine Res 2008;28:351–8. 18593330 10.1089/jir.2007.0056

[18] Al-Mohaya MA Al-Otaibi L Al-Harthi F Association of genetic polymorphisms in interferon-gamma, interleukin-6 and transforming growth factor-beta1 gene with oral lichen planus susceptibility. BMC Oral Health 2016;16:76. 27544215 10.1186/s12903-016-0277-x PMC4992569

[19] Abdel Hay R Rashed L Hegazy R Association of interleukin (IL)18 and IL10 gene polymorphisms with oral lichen planus risk; a case-control study. J Dermatol Sci 2016;83:244–7. 27286581 10.1016/j.jdermsci.2016.05.014

[20] Al-Mohaya MA Al-Harthi F Arfin M TNF-beta and IL-10 gene polymorphism and association with oral lichen planus risk in Saudi patients. J Appl Oral Sci 2015;23:295–301. 26221924 10.1590/1678-775720150075 PMC4510664

[21] Li K Tie H Hu N Association of two polymorphisms rs2910164 in miRNA-146a and rs3746444 in miRNA-499 with rheumatoid arthritis: a meta-analysis. Hum Immunol 2014;75:602–8. 24824381 10.1016/j.humimm.2014.05.002

[22] Srivastava K Srivastava A Comprehensive review of genetic association studies and meta-analyses on miRNA polymorphisms and cancer risk. PloS One 2012;7:e50966. 23226435 10.1371/journal.pone.0050966 PMC3511416

[23] Carrozzo M Dametto E Fasano ME Cytokine gene polymorphisms in hepatitis C virus-related oral lichen planus. Exp Dermatol 2007;16:730–6. 17697145 10.1111/j.1600-0625.2007.00577.x

[24] Bai J Jiang L Lin M Association of polymorphisms in the tumor necrosis factor-alpha and interleukin-10 genes with oral lichen planus: a study in a Chinese cohort with Han ethnicity. J Interferon Cytokine Res 2009;29:381–8. 19450147 10.1089/jir.2008.0089

[25] Xavier GM de Sa AR Guimaraes AL Investigation of functional gene polymorphisms interleukin-1beta, interleukin-6, interleukin-10 and tumor necrosis factor in individuals with oral lichen planus. J Oral Pathol Med 2007;36:476–81. 17686006 10.1111/j.1600-0714.2007.00560.x

[26] Carrozzo M Uboldi de Capei M Dametto E Tumor necrosis factor-alpha and interferon-gamma polymorphisms contribute to susceptibility to oral lichen planus. J Invest Dermatol 2004;122:87–94. 14962095 10.1046/j.0022-202X.2003.22108.x

[27] Tanaka T Narazaki M Kishimoto T IL-6 in inflammation, immunity, and disease. Cold Spring Harb Perspect Biol 2014;6:a016295. 25190079 10.1101/cshperspect.a016295 PMC4176007

[28] Goel S Marwah A Kaushik S Role of serum interleukin-6 in deciding therapy for multidrug resistant oral lichen planus. J Clin Exp Dent 2015;7:e477–482. 26535093 10.4317/jced.52376 PMC4628801

[29] Gu GM Martin MD Darveau RP Oral and serum IL-6 levels in oral lichen planus patients. Oral Surg Oral Med Oral Pathol Oral Radiol Endod 2004;98:673–8. 15583539 10.1016/j.tripleo.2004.05.006

[30] Sun A Chia JS Chang YF Serum interleukin-6 level is a useful marker in evaluating therapeutic effects of levamisole and Chinese medicinal herbs on patients with oral lichen planus. J Oral Pathol Med 2002;31:196–203. 12076322 10.1034/j.1600-0714.2002.310402.x

[31] Xuan Y Wang L Zhi H Association between 3 IL-10 gepe polymorphisms and cardiovascular disease risk: systematic review with meta-analysis and trial sequential analysis. Medicine 2016;95:e2846. 26871859 10.1097/MD.0000000000002846 PMC4753955

