# Supplementary material for: Association between polymorphisms in interleukins and oral lichen planus: A meta-analysis
Source: Medicine (Baltimore). 2017 Mar 24;96(11):e6314. doi: 10.1097/MD.0000000000006314 (PMC5369898; doi:10.1097/MD.0000000000006314)
Supplement: Supplemental Digital Content [file medi-96-e6314-s001.doc]

| Table S1. Scale for methodological quality assessment. | |
| --- | --- |
| **Criteria** | **Score** |
| **1.Representativeness of cases** |  |
| OLP diagnosed according to acknowledged criteria. | 2 |
| Mentioned the diagnosed criteria but not specifically described. | 1 |
| Not Mentioned. | 0 |
| **2.Source of controls** |  |
| Population or community based | 3 |
| Hospital-based OLP-free controls | 2 |
| Healthy volunteers without total description | 1 |
| OLP-free controls with related diseases | 0.5 |
| Not described | 0 |
| **3.Sample size** |  |
| >200 | 2 |
| 80-200 | 1 |
| <80 | 0 |
| **4.Quality control of genotyping methods** |  |
| Repetition of partial/total tested samples with a different method | 2 |
| Repetition of partial/total tested samples with the same method | 1 |
| Not described | 0 |
| **5.Hardy-Weinberg equilibrium** |  |
| Hardy-Weinberg equilibrium in control subjects | 1 |
| Hardy-Weinberg disequilibrium in control subjects | 0 |

OLP=oral lichen planus.

Table S2. Total number of studies related the SNP of ILs

IL=interleukin, SNP=single nucleotide polymorphism.

* These SNP were analysed in this study

| **IL type** | **SNP** | **Number of study** | **Main findings** |
| --- | --- | --- | --- |
| IL1α | -889 T/C | 1 | No significant difference in the genotype and allele were found between OLP patients and controls.[^1^](#_ENREF_1) |
|  | +4845 G/T | 1 | There were no significant differences between the patients and controls in the genotype distributions of +4845 G/T.[^2^](#_ENREF_2) |
| IL1β | +3954 C/T | 2 | No significant difference in the genotype and allele were found between OLP patients and controls. |
|  | -31 C/T | 1 | No significant difference in the genotype and allele were found between OLP patients and controls.[^2^](#_ENREF_2) |
|  | -511C/T | 1 | No significant difference in the genotype and allele were found between OLP patients and controls.[^1^](#_ENREF_1) |
|  | +3962 T/C | 1 | No significant difference in the genotype and allele were found between OLP patients and controls.[^1^](#_ENREF_1) |
| IL1RA | mspa111100 T/C | 1 | No significant difference in the genotype and allele were found between OLP patients and controls.[^1^](#_ENREF_1) |
| IL1R | pst1 1970 C/T | 1 | No significant difference in the genotype and allele were found between OLP patients and controls.[^1^](#_ENREF_1) |
| IL2 | -330 T/G | 2 | No significant difference in the genotype and allele were found between OLP patients and controls. |
|  | +166 G/T | 1 | No significant difference in the genotype and allele were found between OLP patients and controls.[^1^](#_ENREF_1) |
| IL4 | -1098 T/G | 1 | The frequency of the genotype G/G of the IL4 –1098 is more than six fold higher in OLP than in controls[^1^](#_ENREF_1) |
|  | -590 T/C | 2 | No significant difference in the genotype and allele were found between OLP patients and controls. [^1^](#_ENREF_1) Moreover, Bai et al. found that the frequencies of the IL4-590 C allele and the CC genotype in patients with nonerosive OLP were significantly greater than the corresponding values in the control group.[^5^](#_ENREF_5) |
|  | -33 T/C | 1 | No significant difference in the genotype and allele were found between OLP patients and controls. [^1^](#_ENREF_1) |
| IL4RA | -1902 G/A | 1 | No significant difference in the genotype and allele were found between OLP patients and controls. [^1^](#_ENREF_1) |
| IL6 | -174 G/C* | 3 | Please see the article. |
|  | -597 G/A | 1 | There was no significant difference in the allele or genotype frequency distribution between the OLP patients and healthy controls.[^4^](#_ENREF_4) |
|  | -572 C/G | 1 | No significant difference in the genotype and allele were found between OLP patients and controls.[^2^](#_ENREF_2) |
|  | nt565 G/A | 1 | No significant difference in the genotype and allele were found between OLP patients and controls. [^1^](#_ENREF_1) |
| IL8 | −845 T/C | 1 | IL 8 −845 T/C was not present in the sample set and excluded from the further analysis.[^6^](#_ENREF_6) |
|  | −738 T/A | 1 | IL 8 −738 T/A was not present in the sample set and excluded from the further analysis.[^6^](#_ENREF_6) |
|  | −251 A/T | 1 | There was no significant difference in the genotype distribution between OLP patients and the controls. However, AA genotype and A allele frequency was significantly lower in the erosive OLP patients than controls.[^6^](#_ENREF_6) |
|  | +781 C/T | 1 | There was no significant difference in the genotype distribution between OLP patients and the controls.[^6^](#_ENREF_6) |
| IL10 | -819 C/T * | 4 | Please see the article. |
|  | -592 C/A* | 4 | Please see the article. |
|  | -1082 G/A* | 4 | Please see the article. |
|  | -1087 A/G | 1 | No significant difference in the genotype and allele were found between OLP patients and controls.[^2^](#_ENREF_2) |
| IL12A | rs2243115 G/T | 1 | No significant difference in the genotype and allele were found between OLP patients and controls.[^7^](#_ENREF_7) |
|  | rs2243123 C/T | 1 | No significant difference in the genotype and allele were found between OLP patients and controls.[^7^](#_ENREF_7) |
|  | rs583911 A/G | 1 | Higher AA genotype frequencies at position 583911 in the OLP patients compared with healthy controls. However, there was no significant difference in allele frequencies.[^7^](#_ENREF_7) |
|  | rs568408 A/G | 1 | There was a significant difference in rs568408 genotype distributions between patients and controls. Additionally, there were highly significant increases in rs568408 genotype distributions and allele A frequencies in the erosive OLP group as compared to the control group.[^7^](#_ENREF_7) |
|  | rs2243143 A/G | 1 | No significant difference in the genotype and allele were found between OLP patients and controls.[^7^](#_ENREF_7) |
| IL12B | +1188 A/C | 1 | No significant difference in the genotype and allele were found between OLP patients and controls.[^1^](#_ENREF_1) |
| IL18 | -137 G/C | 2 | OLP Patients showed a significant higher percentage of IL18-137 GG genotype and G allele.[^8^](#_ENREF_8) While Bai et al. found no significant difference in the allele or genotype frequency distribution between the OLP patients and healthy controls.[^9^](#_ENREF_9) |
|  | -607 C/A | 2 | OLP Patients showed a significant higher percentage of IL18-607A allele.[^8^](#_ENREF_8) While Bai et al. found that genotype CC and C allele were present at a significantly higher frequency in the OLP patients compared with controls.[^9^](#_ENREF_9) |
|  | -656 G/T | 1 | No significant difference in the allele or genotype frequency distribution between the OLP patients and healthy controls.[^9^](#_ENREF_9) |
|  | 1248 A/G | 1 | No significant difference in the allele or genotype frequency distribution between the OLP patients and healthy controls.[^9^](#_ENREF_9) |

IL=interleukin, SNP=single nucleotide polymorphism.

* These SNP were analysed in this study and the main findings of the included studies please see the article.

**Reference**

1. Carrozzo M, Uboldi de Capei M, Dametto E, et al. Tumor necrosis factor-alpha and interferon-gamma polymorphisms contribute to susceptibility to oral lichen planus. *The Journal of investigative dermatology.* 2004;122(1):87-94.

2. Fujita H, Kobayashi T, Tai H, et al. Assessment of 14 functional gene polymorphisms in Japanese patients with oral lichen planus: a pilot case-control study. *Int J Oral Maxillofac Surg.* 2009;38(9):978-983.

3. Xavier GM, de Sa AR, Guimaraes AL, da Silva TA, Gomez RS. Investigation of functional gene polymorphisms interleukin-1beta, interleukin-6, interleukin-10 and tumor necrosis factor in individuals with oral lichen planus. *Journal of oral pathology & medicine : official publication of the International Association of Oral Pathologists and the American Academy of Oral Pathology.* 2007;36(8):476-481.

4. Chauhan I, Beena VT, Srinivas L, Sathyan S, Banerjee M. Association of cytokine gene polymorphisms with oral lichen planus in Malayalam-speaking ethnicity from South India (Kerala). *Journal of interferon & cytokine research : the official journal of the International Society for Interferon and Cytokine Research.* 2013;33(8):420-427.

5. Bai J, Lin M, Zeng X, et al. Association of polymorphisms in the human IFN-gamma and IL-4 gene with oral lichen planus: a study in an ethnic Chinese cohort. *Journal of interferon & cytokine research : the official journal of the International Society for Interferon and Cytokine Research.* 2008;28(6):351-358.

6. Dan H, Liu W, Zhou Y, Wang J, Chen Q, Zeng X. Association of interleukin-8 gene polymorphisms and haplotypes with oral lichen planus in a Chinese population. *Inflammation.* 2010;33(2):76-81.

7. Jiang C, Yao H, Cui B, Zhou Y, Wang Y, Tang G. Association of interleukin 12A gene polymorphisms with oral lichen planus in Chinese population. *Journal of oral pathology & medicine : official publication of the International Association of Oral Pathologists and the American Academy of Oral Pathology.* 2015;44(8):602-606.

8. Abdel Hay R, Rashed L, Hegazy R, Rashwan W, Samir N, Nour-Edin F. Association of interleukin (IL)18 and IL10 gene polymorphisms with oral lichen planus risk; a case-control study. *Journal of dermatological science.* 2016;83(3):244-247.

9. Bai J, Zhang Y, Lin M, et al. Interleukin-18 gene polymorphisms and haplotypes in patients with oral lichen planus: a study in an ethnic Chinese cohort. *Tissue antigens.* 2007;70(5):390-397.

| **Study** | **Representativeness of cases** | **Source of controls** | **Sample size** | **Quality control of genotyping methods** | **HWE** | **Total Score of quality assessment scale** |
| --- | --- | --- | --- | --- | --- | --- |
|
| Al-Mohaya MA 2016 | 2 | 1 | 2 | 0 | 1 | 6 |
| Abdel Hay R 2016 | 2 | 2 | 1 | 0 | 0 | 5 |
| Al-Mohaya MA 2015 | 2 | 3 | 2 | 0 | 0 | 7 |
| Bai 2009 | 1 | 1 | 2 | 1 | 0 | 5 |
| Xavier GM 2007 | 2 | 2 | 1 | 0 | 0 | 5 |
| Carrozzo M 2004 | 2 | 3 | 1 | 1 | 0 | 7 |

Table S3 Methodological quality of the included studies

HWE：Hardy–Weinberg equilibrium
